# Cervical Local Cytokine Release Syndrome Following Chimeric Antigen Receptor T-cell Therapy in Patients With Relapsed or Refractory Diffuse Large B-cell Lymphoma

**DOI:** 10.7759/cureus.38905

**Published:** 2023-05-11

**Authors:** Yu Inoue, Takahiro Fujino, Shotaro Chinen, Yui Niiyama-Uchibori, Daisuke Ide, Moe Kawata, Keiko Hashimoto, Tomoko Takimoto-Shimomura, Ai Nakayama, Taku Tsukamoto, Shinsuke Mizutani, Yuji Shimura, Shigeru Hirano, Junya Kuroda

**Affiliations:** 1 Division of Hematology and Oncology, Department of Medicine, Kyoto Prefectural University of Medicine, Kyoto, JPN; 2 Department of Otolaryngology-Head and Neck Surgery, Kyoto Prefectural University of Medicine, Kyoto, JPN; 3 Department of Hematology, Otsu City Hospital, Otsu, JPN

**Keywords:** local cytokine release syndrome, diffuse large b-cell lymphoma, chimeric antigen receptor t-cell therapy, dexamethasone, tocilizumab

## Abstract

The use of chimeric antigen receptor T-cell (CAR-T) therapy for hematologic malignancies is rapidly increasing, and appropriately managing adverse events (AEs) is crucial. Cytokine release syndrome (CRS) is a common AE of CAR-T therapy, characterized by systemic symptoms such as fever and respire-circulatory failure. We present two cases with relapsed or refractory diffuse large B-cell lymphoma (DLBCL) accompanied by a rare complication of cervical local CRS as an acute inflammatory reaction at a specific site after CAR-T infusion. Case 1: A 60-year-old gentleman with diffuse large B cell lymphoma (DLBCL) developed grade 1 CRS on day one that required three doses of tocilizumab. Then he developed remarkable cervical edema as local CRS on day five. His local CRS spontaneously improved from day seven without additional therapy. Case 2: A 70-year-old gentleman with DLBCL developed grade 1 CRS on day two that required three doses of tocilizumab. Then he developed remarkable cervical edema and muffled voice as local CRS on day three. He received dexamethasone because of concerns about airway obstruction, and his local CRS improved immediately after dexamethasone administration. Before Tisa-Cel infusion, neither patients had a lymphoma lesion in their necks. To summarize, local CRS may occur at the site without lymphoma involvement after CAR-T therapy. An appropriate diagnosis and careful observation are required to determine the need for additional treatment.

## Introduction

Anti-CD19 chimeric antigen receptor T-cell (CAR-T) therapy has shown remarkable efficacy in treating patients with hematologic malignancies, particularly B-cell malignancies resistant to currently available immunochemotherapy [1,2]. Despite their efficacy, CAR-T therapy may cause various adverse events (AEs), such as cytokine release syndrome (CRS), neurologic complications named immune effector cell-associated neurotoxicity syndrome (ICANS), cytopenias, and hypogammaglobulinemia [3-9]. CRS is one of the most common AEs associated with CAR-T therapy due to massive cytokine release. It is characterized by systemic symptoms, such as pyrexia and occasional circulatory/respiratory failure, mostly in the early post-CAR-T cell infusion. Compared to these prevalent systemic symptoms of CRS, an acute inflammatory phenomenon at specific body regions, named local CRS, is a rare complication. However, data on local CRS is scarce [10-13]. We here present two patients who developed local CRS with significant cervical edema following anti-CD19 CAR-T therapy for relapsed or refractory diffuse large B-cell lymphoma (DLBCL).

## Case presentation

Case 1

**Figure 1:**
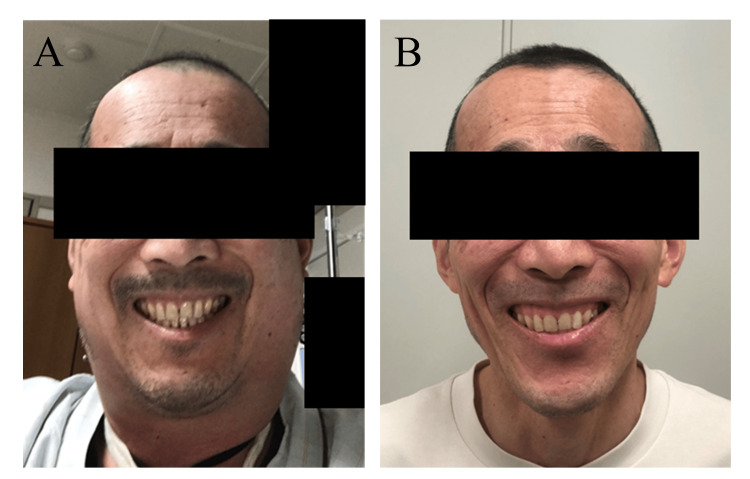
Gross appearance of cervical swelling in Case 1. A. At the emergence of local cytokine release syndrome (CRS) on day five post-CAR-T cell infusion. B. After the resolution of local CRS.

The patient was a 60-year-old male diagnosed with DLBCL. 18F-fluorodeoxyglucose (FDG) positron emission tomography combined with computed tomography (PET-CT) revealed the presence of multiple FDG-avid lesions systemically, including systemic enlarged lymph nodes, the intestinal tract, liver, and right iliac bone, which leads to stage IV according to the Ann Arbor staging system. At the initial presentation, he had no cervical lesions. While the initial therapy with R-CHOP (rituximab, cyclophosphamide, vincristine, doxorubicin, and prednisolone) and the first-salvage therapy with CHASER (rituximab, cyclophosphamide, cytarabine, and etoposide) did not induce remission, the second salvage therapy by two cycles of Pola-BR (polatuzumab vedotin, bendamustine, and rituximab) with concurrent radiation therapy for the right iliac bone lesion induced the partial metabolic response (PMR) without cervical lesion. Then, the patient undertook anti-CD19 CAR-T cell therapy using Tisa-Cel for the primary refractory DLBCL. The patient experienced grade 1 CRS with pyrexia over 38 ℃ the day following the infusion of Tisa-Cel, necessitating treatment with 8mg/kg of tocilizumab three times during days three to four. Meanwhile, his respiratory and circulatory status was stable. However, on day five, the patient suddenly developed significant swelling in his neck without dyspnea or stridor (Figure 1A).

**Figure 2:**
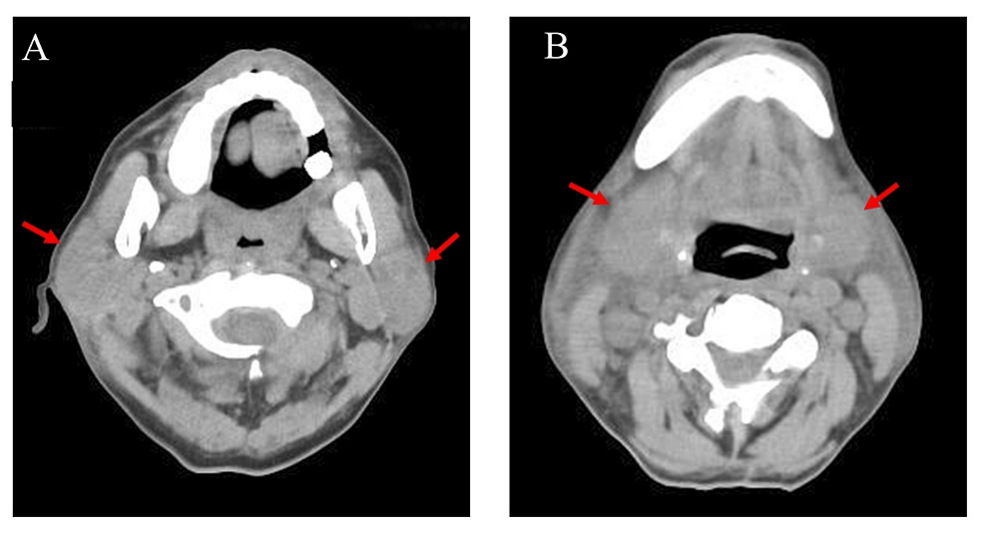
Plain computed tomography scan of the neck on day six post-chimeric antigen receptor T-cell (CAR-T) cell infusion in Case 1. A. Swelling of bilateral parotid glands (red arrows). B. Swelling of submaxillary glands (red arrows).

Blood tests revealed pancytopenia with leukocyte counts of 1.0 × 109/L, consisting of 35% of neutrophils, 41% lymphocytes, 10% monocytes, and 14% eosinophils, but no lymphoma cells, 9.1 g/dL of hemoglobin, and 80.0 × 109/L of platelets count. The serological tests showed slightly elevated C-reactive protein (CRP) to 1.21 mg/dL. There were no other prominent abnormalities, including lactate dehydrogenase and mumps IgM. The plain CT revealed remarkable subcutaneous edema and the swelling of bilateral parotid and submandibular glands (Figure 2).

We determined that the patient had local CRS in his salivary glands and surrounding tissues. Day seven marked the beginning of his fever's gradual improvement without using corticosteroids, as well as the neck swelling. He was released on day 19, and three months after Tisa-Cel, his PET-CT revealed a complete metabolic response (CMR). Also, his cervical edema has recovered to the baseline (Figure 1B).

Case 2

**Figure 3:**
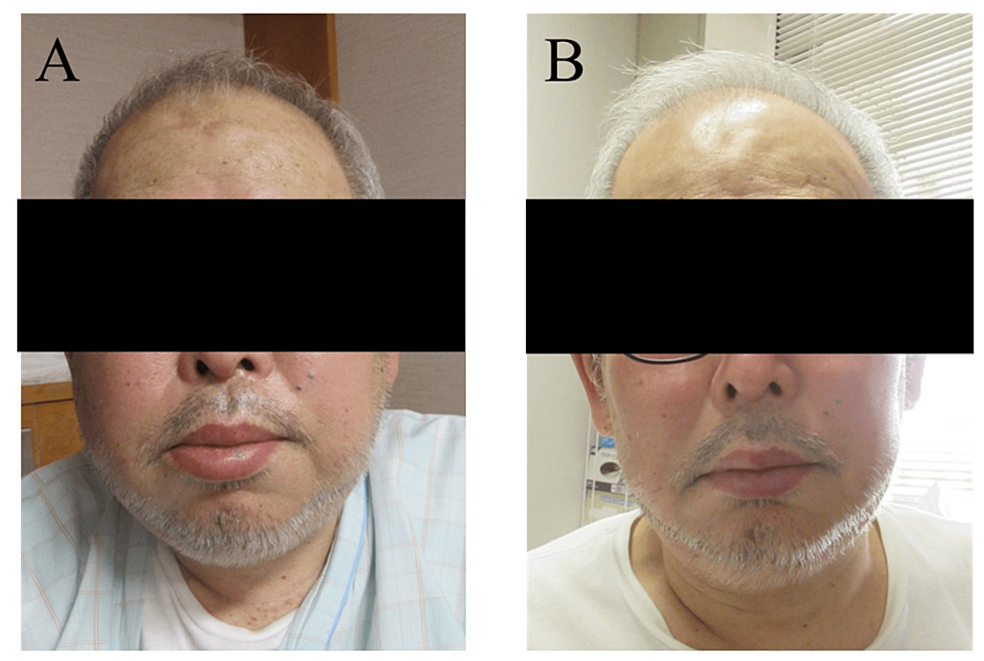
Gross appearance of cervical swelling in Case 2. A. At the emergence of local cytokine release syndrome (CRS) on day four post-CAR-T cell infusion. B. The day after administration of dexamethasone (day five).

The patient was a 70-year-old male diagnosed with DLBCL, stage III according to the Ann Arbor staging system, having systemically enlarged lymph nodes, including those at the neck. Despite the transient CMR induced by six cycles of R-CHOP, the disease relapsed seven months later with enlarged perihilar lymph nodes and abdominal fluid involvement. After the achievement of partial response by the salvage chemotherapy by four cycles of R-GCD (gemcitabine, carboplatin, dexamethasone, and rituximab), the patient was subjected to the anti-CD19 CAR-T cell therapy using Tisa-Cel. He had no cervical lesions just before CAR-T therapy. He experienced grade 1 CRS on the day of Tisa-Cel infusion, necessitating treatment with 8mg/kg of tocilizumab three times from day two. On day three, the patient suddenly developed remarkable cervical swelling and a muffled voice, and these symptoms worsened on day four (Figure 3A).

**Figure 4:**
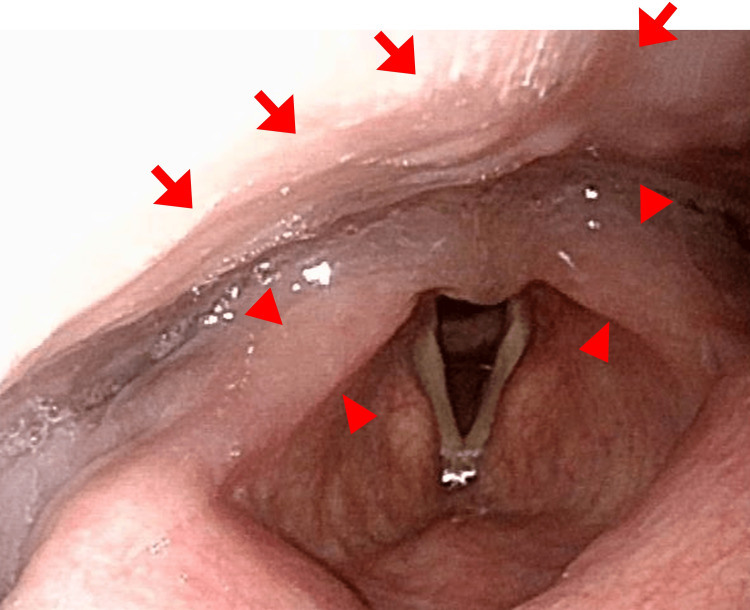
Laryngoscopy just after dexamethasone administration. The posterior pharyngeal wall (red arrows) and laryngeal arytenoid (red arrowheads) were swollen.

Blood tests at this point revealed anemia with 8.2 g/dL of hemoglobin, thrombocytopenia with 111.0 × 109/L of platelets counts, and a slight decrease of leukocyte counts of 3.4 × 109/L, consisting of 85% of neutrophils, 6% lymphocytes, 7% monocytes, and 2% eosinophils. The serological tests showed the elevation of CRP to 4.21 mg/dL without other prominent abnormalities. Laryngoscopy showed that the posterior pharyngeal wall and laryngeal arytenoid were swollen (Figure 4).

We determined that the patient had local CRS in his neck. We emergently administered 9.9 mg of dexamethasone for dyspnea, and, then, his symptom immediately resolved. Several hours after the administration of dexamethasone, his neck swelling and muffled voice improved (Figure 3B), and he was released on day 19.

## Discussion

In contrast to systemic CRS, local CRS is uncommon and has only been reported sporadically in patients receiving CAR-T therapy for various malignancies, including ovarian cancer, B-cell lymphoma, and B-cell acute lymphocytic leukemia (B-ALL) [10-13]. However, the local CRS's precise mechanism has not been fully elucidated.

Systemic CRS is said to develop, expand, and resolve through several phases with distinct kinetics of CAR-T cells after infusion; the initial infiltration and expansion of CAR-T cells in tumor masses, the subsequent systemic overflow of CAR-T cells leading to systemic inflammation, CAR-T cell redistribution leading to organ damage, and the resolution phase [3,10]. According to these CRS models, local CRS can occur in two ways. The first is that the initial local accumulation and expansion of CAR-T cells in tumor masses causes local CRS via a localized severe immune response. The case of local CRS was reported in patients with primary cervical tumor lesions, which may support this hypothesis [12]. The other possibility is that local CRS is attributed to localized acute inflammation caused by CAR-T cell redistribution to non-tumorous tissues. Indeed, there are reports of local CRS occurring in non-tumorous cervical areas, such as our cases, implying a link between local CRS and an abundance of normal lymphoid tissues in the cervical area [10,11]. Furthermore, local CRS was preceded by systemic CRS in our cases. These findings may indicate that the scenario underlying our cases was more consistent with the latter model due to the redistribution of CAR-T cells following systemic overflow.

While tocilizumab, an interleukin-6 (IL-6) receptor antagonist, is one of the most important therapeutic modalities for systemic CRS [3-9], its effectiveness for local CRS is not elucidated [10-12]. In our cases, tocilizumab for systemic CRS did not prevent the development of subsequent local CRS. In contrast, previous reports suggested the utility of corticosteroids against the local CRS [10-12]. In Case 1, we did not use corticosteroids because his airway was not obstructed, and his systemic and local CRS improved without additional treatment except tocilizumab. However, in Case 2, he had a muffled voice and received dexamethasone because of concerns about airway obstruction, and his systemic and local CRS improved immediately after dexamethasone administration. Careful observation is required to use corticosteroids for the cervical local CRS when there is a sign of airway obstruction.

## Conclusions

In conclusion, it is crucial to consider local CRS as a rare and novel therapy-related pathologic condition of cervical swelling during CAR-T therapy, even in patients without primary lymph node lesions around the neck. In addition, tocilizumab may be insufficient for local CRS, and therapeutic decision-making using corticosteroids is crucial to avoid life-threatening airway obstruction.
